# The role of momentum-dark excitons in the elementary optical response of bilayer WSe_2_

**DOI:** 10.1038/s41467-018-04877-3

**Published:** 2018-07-03

**Authors:** Jessica Lindlau, Malte Selig, Andre Neumann, Léo Colombier, Jonathan Förste, Victor Funk, Michael Förg, Jonghwan Kim, Gunnar Berghäuser, Takashi Taniguchi, Kenji Watanabe, Feng Wang, Ermin Malic, Alexander Högele

**Affiliations:** 1 Fakultät für Physik, Munich Quantum Center, and Center for NanoScience (CeNS), Ludwig-Maximilians-Universität München, Geschwister-Scholl-Platz 1, D-80539 München, Germany; 2 Department of Physics, Chalmers University of Technology, SE-412 96 Gothenburg, Sweden; 3 Institut für Theoretische Physik, Nichtlineare Optik und Quantenelektronik, Technische Universität Berlin, Hardenbergstr. 36, D-10623 Berlin, Germany; 4 Department of Physics, University of California at Berkeley, Berkeley, CA 94720 USA; 5 National Institute for Materials Science, Tsukuba, Ibaraki 305-0044 Japan

## Abstract

Monolayer transition metal dichalcogenides (TMDs) undergo substantial changes in the single-particle band structure and excitonic optical response upon the addition of just one layer. As opposed to the single-layer limit, the bandgap of bilayer (BL) TMD semiconductors is indirect which results in reduced photoluminescence with richly structured spectra that have eluded a detailed understanding to date. Here, we provide a closed interpretation of cryogenic emission from BL WSe_2_ as a representative material for the wider class of TMD semiconductors. By combining theoretical calculations with comprehensive spectroscopy experiments, we identify the crucial role of momentum-indirect excitons for the understanding of BL TMD emission. Our results shed light on the origin of quantum dot formation in BL crystals and will facilitate further advances directed at opto-electronic applications of layered TMD semiconductors in van der Waals heterostructures and devices.

## Abstract (web summary)

The electronic band structure of atomically thin transition metal dichalcogenides is strongly sensitive to the number of layers, resulting in modified light emission. Here, the authors investigate the cryogenic emission from bilayer WSe_2_ to identify the role of momentum-indirect excitons for its optical response.

## Introduction

Semiconductor TMDs exhibit remarkable properties in the monolayer (ML) limit, including a direct bandgap at the K and K′ points of the hexagonal Brillouin zone (BZ)^1,2^ with unique spin and valley physics^3^ of value for novel opto-valleytronic applications^4–8^. In addition to bright excitons^9–11^ composed of electrons and unoccupied states in K (or K′) valleys with collinear out-of-plane spin projections, tungsten-based MLs feature lowest-lying spin-dark excitons^12–15^ as combinations of K (or K′) states of opposite spin^16–21^. The realm of both momentum-direct excitons is expanded by the notion of momentum-indirect excitons involving electrons and unoccupied states in different valleys^22–28^. In ML WSe_2_, for example, momentum-indirect excitons can be constructed from conduction and valence band states in opposite K and K′ valleys^23,26,28^, involve unoccupied states in the Γ valley^27^, or electrons in Q pockets that reside roughly halfway between the Γ and K (or K′) points of the first BZ^26^.

In BL TMDs, the single-particle bandgap is indirect because of a downshift of the conduction band (CB) energy at Q well below K and an upshift of the valence band (VB) edge at the Γ point upon the addition of a second layer^1,29–32^. In the specific case of BL WSe_2_ crystals, the lowest CB minimum is located at Q, while the VB maximum at K exceeds the one at Γ only by 40 ± 30 meV according to angle-resolved photoemission spectroscopy^33^. The associated photoluminescence (PL) spectra are thus dominated by momentum-indirect transitions interconnecting CB electrons and empty VB states in dissimilar valleys^30,34–40^. The BL emission is consistently less efficient, with PL from short-lived direct excitons^39^ redshifted by a few tens of meV from the ML peak emission, and a second peak with larger redshift and longer lifetimes^39^ attributed to momentum-indirect excitons composed of electrons in the K or Q valleys and unoccupied states in the K or Γ valleys^36–40^. A detailed understanding of both peaks, however, has remained elusive^41^ despite the significance of BL TMDs as hosts of novel single-photon sources^42,43^, finite valley polarization^39^, or potential utilization of the spin-layer locking effect in charged BLs^44^.

Here, we present a comprehensive study of exciton manifolds in BL WSe_2_ carried out both in experiment and theory. Using cryogenic spectroscopy of BL regions subjected to strain at unintentional disorder, we identify brightening of momentum-indirect excitons that in some cases is accompanied by the formation of quantum dots (QDs) with intense emission of non-classical light. Complementary experiments reveal the energy-level hierarchy of all excitons involved in determining the optical response of BL WSe_2_. These findings, in good quantitative agreement with theoretical calculations, not only explain the intricate details of the BL PL spectra and the origin of the QD PL, they can be also generalized to other representatives of TMD materials to facilitate a detailed understanding of opto-electronic properties of BL and multilayer semiconductors.

## Results

### Photoluminescence spectroscopy of bilayer WSe_2_

**Fig. 1:**
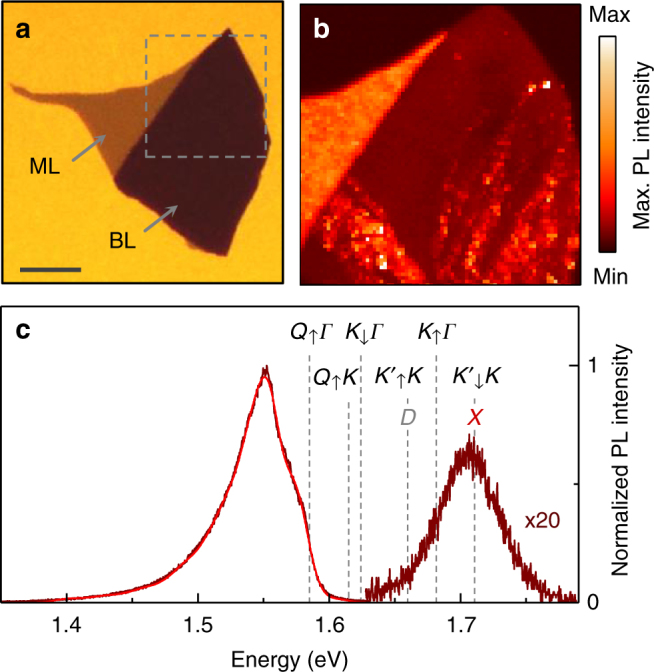
Photoluminescence spectroscopy of bilayer WSe_2_. **a** Optical micrograph of a WSe_2_ flake exfoliated onto Si/SiO_2_ with monolayer (ML) and bilayer (BL) regions indicated by the arrows (scale bar, 15 μm). **b** Cryogenic raster-scan photoluminescence map of the upper corner indicated by the dashed square in **a**. False-color plot of the photoluminescence maxima in the spectral range of 1.43–1.59 eV. The bilayer exhibits extended and punctual regions of brightening attributed to strain at local folds. **c** Normalized photoluminescence spectrum (brown, magnified by a factor of 20 in the range of 1.62–1.82 eV) at a representative bilayer position away from defects with model fit shown as red solid line. The energy positions of momentum-bright (*X* and *D*) and momentum-dark BL excitons (*Q*_↑_*Γ*, *Q*_↑_*K*, *K*_↓_*Γ*, $K'_ \uparrow K$, *K*_↑_*Γ*, and $K'_ \downarrow K$, labeled by the capital letters of electron and empty state valleys and the electron out-of-plane spin as subscript) are indicated by dashed lines. All spectroscopy measurements were performed at 4.2 K with excitation at 1.95 eV

Cryogenic spectroscopy of ML and BL WSe_2_ was carried out on a flake shown in Fig. 1a obtained by standard exfoliation onto an Si/SiO_2_ substrate (see Supplementary Notes 1 and 2 for details). Extended ML and BL regions (marked with arrows) were identified by their respective contrast in the optical micrograph of Fig. 1a and by Raman spectroscopy. The dashed square indicates the region of the cryogenic hyperspectral raster-scan PL map recorded with a home-built confocal microscope. The false-color map in Fig. 1b shows PL peak maxima in the spectral range of 1.43–1.59 eV, highlighting extended homogeneous regions of bright ML and dim BL luminescence, as well as distinct BL regions of unintentional disorder with PL brightening due to local strain^42,43^.

A characteristic PL spectrum of BL WSe_2_ on SiO_2_, recorded at 4.2 K on a representative position away from disorder, is shown in Fig. 1c (see Supplementary Fig. 1 for spectra from other BL WSe_2_ samples). The PL exhibits a weak peak around 1.71 eV and a stronger peak around 1.55 eV consistent with previous PL studies of BL WSe_2_^36,38–40^. Based on comprehensive experiments described in the following, we develop a model to interpret the PL of WSe_2_ BLs as originating from both momentum-direct and momentum-indirect excitons with energy positions indicated by the dashed lines in Fig. 1c.

### Excitons in bilayer WSe_2_

**Fig. 2:**
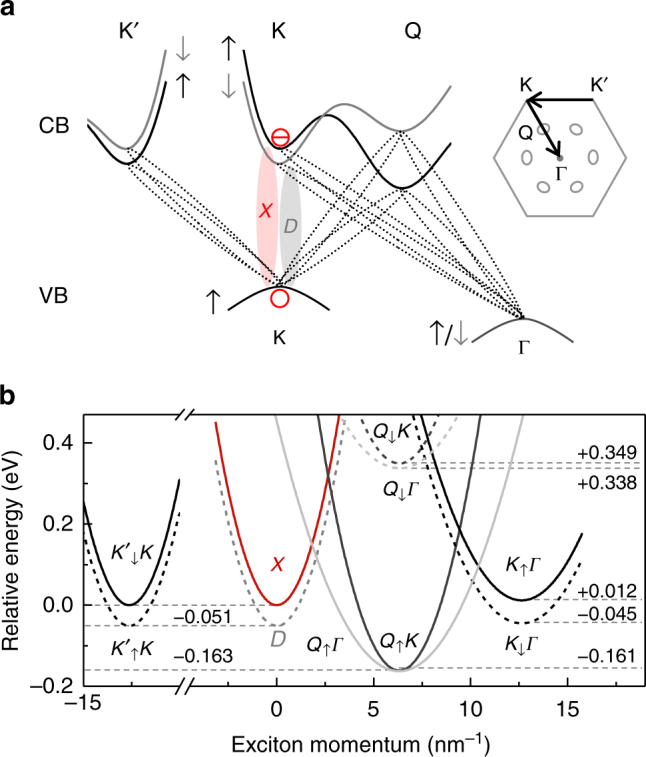
Excitons in bilayer WSe_2_. **a** Schematic single-particle band diagram of the conduction and the valence bands of bilayer WSe_2_ along high-symmetry lines of the hexagonal Brillouin zone shown on the right. Zero-momentum spin-bright (*X*) and spin-dark (*D*) excitons are formed in the K valley by electrons from spin-up and spin-down conduction sub-bands indicated in black and gray, respectively, paired with a spin-up valence band empty state. Momentum-indirect excitons with electrons and unoccupied states in dissimilar valleys are indicated by dashed ellipses. **b** Calculated dispersions of lowest-energy exciton manifolds in bilayer WSe_2_ with energy minima given in eV with respect to the bright exciton *X*

To identify all relevant excitons that contribute to cryogenic PL and to interpret the model fit to the lower-energy PL peak shown as the red solid line in Fig. 1c, it is instructive to consider first the single-particle band structure of BL WSe_2_ in Fig. 2a and the associated exciton dispersions plotted in Fig. 2b. The relevant states for the construction of excitons (indicated by ellipses in Fig. 2a) with an empty state located at the VB maxima in the K or Γ valley are the spin-polarized sub-band minima near K, Q, and K′ valleys of the CB, with out-of-plane spin projections indicated by the arrows. We take the spin degenerate VB maximum at Γ to be 40 meV below the energy of the spin-polarized band edge at K^33^, and the energies of the CB at K, Q, and K′ from density functional theory calculations^45,46^.

The excitonic dispersions, shown in Fig. 2b, were computed using the Wannier equation^47,48^ within the Keldysh formalism^49–51^, taking explicitly the dielectric environment of the TMD material into account. The corresponding excitons, all of which have their counterparts with the empty VB state at K′, can be separated into the class of zero-momentum excitons with spin-allowed and spin-forbidden configuration (labeled as *X* and *D*, respectively), and finite-momentum excitons involving Coulomb-correlated electrons and unoccupied VB states from dissimilar valleys (labeled in Fig. 2b according to the valleys of the CB electron and the VB unoccupied state as capital letters with the electron out-of-plane spin as subscript). All excitons but *X* are dipole-forbidden, either due to spin or momentum conservation constraints.

Energy minima of the branches are given in eV with respect to the bright exciton *X* (see Supplementary Note 3 for the details of theoretical calculations). Consistent with the downshift (upshift) of the Q (Γ) valley in the CB (VB) of BL WSe_2_, we found the smallest exciton gap for finite-momentum *Q*_↑_*Γ* and *Q*_↑_*K* excitons, followed by six branches involving an electron in K or K′ (two energy degenerate branches of *D* and $K_ \uparrow ^\prime K$, and *X* and $K_ \downarrow ^\prime K$ excitons with the unoccupied state at K, as well as exciton branches *K*_↑_*Γ* and *K*_↓_*Γ* with the empty VB state at Γ), and two branches of excitons at highest energies with electrons in one of the three spin-down polarized Q valleys forming *Q*_↓_*Γ* and *Q*_↓_*K* with the unoccupied states in Γ and K valleys, respectively. We emphasize that although we use a spin-tagged notation for Q-momentum excitons consistent with the schematics in Fig. 2a, there are energetically degenerate excitons composed of electrons from the other three inequivalent Q pockets with opposite spin.

Out of this set of excitons, spin-bright *X* states emit PL along the detection axis of our microscope, and the PL from spin-dark *D* excitons with in-plane emission is detected due to the high numerical aperture of the objective as well^14^. In contrast, all momentum-indirect excitons appear exclusively as phonon replicas of their optically dark zero-phonon line (ZPL) as they emit photons only with the assistance of acoustic or optical phonons. With this constraint in mind, we note that the higher-energy peak of the BL spectrum in Fig. 1c is dominated by the ZPL of *X* (in accord with the onset of differential reflectivity, shown in Supplementary Fig. 1a) with a weak contribution from *D* to the red wing, while the lower-energy PL peak is a superposition of phonon sidebands of momentum-dark excitons *Q*_↑_*Γ*, *Q*_↑_*K*, and *K*_↓_*Γ*.

Postponing a detailed explanation for the energy ladder of all relevant exciton states indicated by the dashed lines in Fig. 1c, we first discuss the model fit of the lower-energy peak in the BL spectrum. For the decomposition of the peak (red solid line in Fig. 1c) into the PL contributions from *Q*_↑_*Γ*, *Q*_↑_*K*, and *K*_↓_*Γ*, we set the energy positions of the respective dark ZPLs to the experimentally determined values and modeled the phonon replicas by inhomogeneously broadened Gaussians with a full-width at half-maximum linewidth *γ*. For simplicity, we involved only one branch of acoustic and optical phonons (the longitudinal acoustic and optical phonon branch) with energies given in ref. ^52^ (see Supplementary Table 1 for phonon energies and Supplementary Note 4 for details of the fit procedure). Best fit to the spectrum was obtained with the inhomogeneous linewidth *γ* = 21 meV. The inclusion of up to sixth-order scattering processes was required to reproduce the extended low-energy tail of the spectrum.

At the level of theory, the energetic ordering of *Q*_↑_*Γ* and *Q*_↑_*K* states is ambiguous given the small difference of 2 meV in the energy minima of the two branches (Fig. 2b). However, complementary spectroscopy experiments on strained BL regions and QDs discussed in the following remove this ambiguity and establish the energy scale hierarchy for all excitons responsible for the elementary optical response of BL WSe_2_ with *Q*_↑_*Γ* as the lowest-energy exciton branch, followed by *Q*_↑_*K*, *K*_↓_*Γ*, degenerate *D* and $K_ \uparrow ^\prime K$ states, *K*_↑_*Γ*, and degenerate *X* and $K_ \downarrow ^\prime K$ manifolds.

### Effects of strain and local disorder

**Fig. 3:**
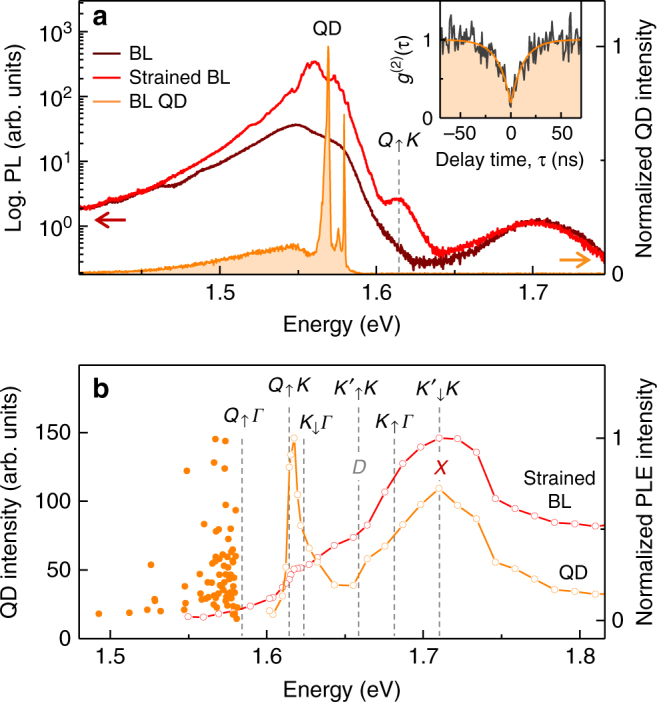
Effects of strain and localization on the photoluminescence of bilayer WSe_2_. **a** Photoluminescence from a strained bilayer region without (red) and with (orange) spectrally narrow and intense quantum dot (QD) emission recorded at a factor of 1000 lower-excitation power. The bilayer spectrum away from strained regions (brown) is shown for reference. Note the strain-induced emergence of the shoulder at 1.615 eV labeled as *Q*_↑_*K*. Inset: typical second-order coherence of a single quantum dot with pronounced antibunching on ~10 ns timescale. **b** Distribution of quantum dot intensities as a function of their peak emission energies (filled circles, extracted from the map of Fig. 1b), and photoluminescence excitation spectra of the quantum dot and strained bilayer emission in **a** (orange and red open circles, respectively). The dashed lines mark the energy positions of the relevant exciton states

The first input to the experimental determination of the exciton energies is provided by the PL spectroscopy of QDs distributed randomly along the lines of disorder as in Fig. 1b. BL QDs, with intense and spectrally narrow PL emission as in Fig. 3a, emerge as a result of local strain^42,43^. Akin to ML QDs^42,53–58^, the QDs in disordered BL regions were characterized by strong antibunching signatures in the second-order correlation function *g*^(2)^(*τ*) of their PL emission^42,43^, as demonstrated exemplarily by the inset data of Fig. 3a recorded on a different QD with a dip of 0.2 at *τ* = 0 and an exponential rise to 1 on a timescale of ~10 ns. By plotting the PL intensity as a function of the respective energy maximum for all QDs of the hyperspectral map of Fig. 1b, we identify a sharp cutoff to the QD emission energy at 1.584 eV (indicated by the leftmost dashed line in Fig. 3b), which we assign to the state *Q*_↑_*Γ* (see Supplementary Note 5 and Supplementary Fig. 2 for assignment).

The energy position of the next higher-energy momentum-dark state is revealed by the PL spectroscopy of strained BL regions. The PL spectrum on a strained position features characteristic blue and redshifts of a few meV for the upper and lower PL peaks (compare red and brown traces in Fig. 3a) consistent with ~0.1% of tensile strain which lowers (raises) the CB energy minimum at K (Q)^59^. In addition, a shoulder at 1.615 eV, indicated by the dashed line in Fig. 3a, becomes apparent due to strain-induced brightening of this momentum-dark transition^60^. The energy position of this shoulder reappears as a resonance in the photoluminescence excitation (PLE) spectrum of a strained BL spot (open red circles in Fig. 3b). The resonance, marked by the dashed line and assigned to *Q*_↑_*K*, is even more pronounced in the PLE spectrum of the QD from the same spot position (with the spectrum in Fig. 3a) shown by open orange circles in Fig. 3b. We note that the PLE spectrum is not QD specific, it rather represents generic BL resonances in the PLE of QDs emitting at different observation sites with different energies (see Supplementary Fig. 3 for PLE spectra of other QDs).

The third successive energy level of momentum-dark states, identified at 1.624 eV by the resonance and the shoulder of the QD and strained BL PLE spectra of Fig. 3b, respectively, is ascribed to *K*_↓_*Γ*. With this energy, the experimental values of the three lowest-energy momentum-dark exciton states can now be hierarchically ordered with respect to the energy of the bright exciton *X* at 1.710 eV deduced from the peaks of both PLE spectra of Fig. 3b and from PL in Fig. 1c (and differential reflectivity shown in Supplementary Fig. 1a). Referencing all energies to that of *X*, we note first that the lowest momentum-forbidden state *Q*_↑_*Γ* is redshifted by 126 meV instead of the calculated value of 163 meV, while the second lowest state *Q*_↑_*K* exhibits a redshift of 95 meV instead of 161 meV expected from theory. Provided that the effective masses used in the calculations of exciton energies were correct, these quantitative discrepancies between theory and experiment convert into an upshift of the CB minimum at the Q valley by 66 meV and a downshift of the VB at the Γ point by 29 meV. Given the uncertainties in band structure calculations^45,46^ and angle-resolved photoemission^33^ used to calculate the exciton dispersion minima, these corrections of a few tens of meV seem reasonable.

Finally, with the energies of *X* and *K*_↓_*Γ* at hand, we estimate the energies of *D* and *K*_↑_*Γ* in Fig. 3b by using the respective spin–orbit splittings of 51 and 57 meV from Fig. 2b. While the energy level of *K*_↑_*Γ* has no compelling signature in the PLE spectra of Fig. 3b, the *D* state coincides with a clearly pronounced shoulder in the PLE spectrum of the QD. To complete the energetic ordering of all lowest-lying excitons in BL WSe_2_, the states $K_ \uparrow ^\prime K$ and $K_ \downarrow ^\prime K$ are placed in resonance with *D* and *X* by omitting electron–hole exchange.

### Field-effect control of doping in bilayer WSe_2_ embedded in hexagonal boron nitride

**Fig. 4:**
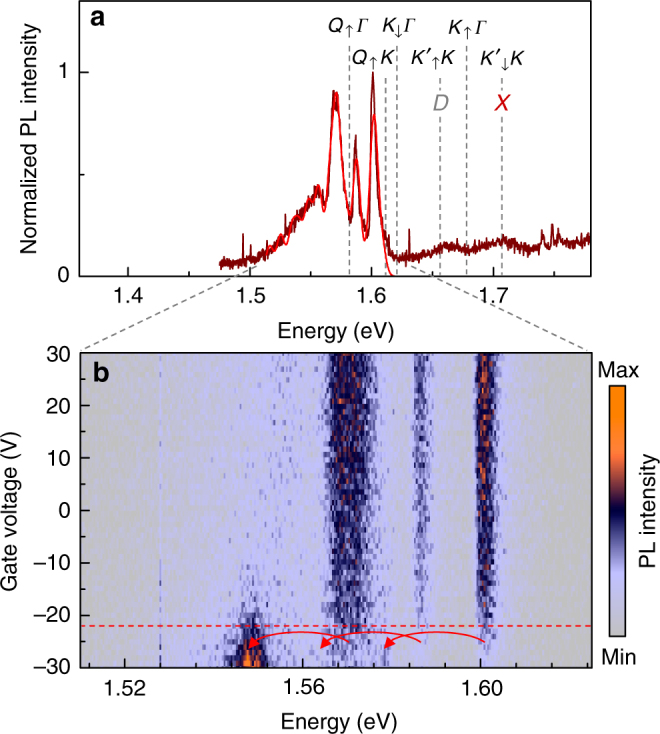
Field-effect control of bilayer WSe_2_ photoluminescence. **a** Normalized photoluminescence (brown) of a bilayer WSe_2_ encapsulated in hexagonal boron nitride and tuned toward charge neutrality with positive gate voltages. Best fit to the spectrum (red) was obtained with the same set of excitons energies as in Fig. 1c and an overall redshift of 2 meV. **b** Evolution of the photoluminescence with gate voltage from −30 to 30 V. Note the cross-over to the charged regime below −20 V signified by a simultaneous redshift of 22 meV for all peaks as indicated by the red arrows below the dashed line

We tested this set of exciton energies obtained from the analysis of PL signatures of BL WSe_2_ on SiO_2_ in Fig. 1c on narrow spectra of a gate-tunable WSe_2_ bilayer encapsulated in hexagonal boron nitride (hBN). Figure 4a shows the normalized PL spectrum (brown) under charge neutrality conditions with the corresponding model fit. Remarkably, the intricate spectral features of the spectrum can be reproduced with the set of exciton energies established above with an overall redshift of 2 meV. The spectrally narrow inhomogeneous linewidths down to ~8 meV required a refined model fit with all phonon modes included and variations of ±2 meV around the values calculated for ML WSe_2_ in ref. ^52^ (see Supplementary Note 4 for details of the fit procedure).

Including first-order scattering from *K*_↓_*Γ* and *Q*_↑_*K* exciton manifolds, and up to second-order scattering from *Q*_↑_*Γ*, our model identifies the blue-most peak at ~1.6 eV out of the three strong peaks in the spectrum of Fig. 4a in the range 1.61–1.57 eV as optical and acoustic phonon replica of *K*_↓_*Γ* and *Q*_↑_*K*, respectively, the central peak as an optical sideband of *Q*_↑_*K*, and the red-most peak as an acoustic sideband of *Q*_↑_*Γ*. The consecutive peak toward lower energies around 1.56 eV is an optical sideband of *Q*_↑_*Γ* that merges with the extended red wing composed of higher-order phonon replica from all the above-mentioned momentum-dark states.

Interestingly, the ambiguity with respect to the origin of the blue-most peak as being composed of acoustic or optical sidebands of *K*_↓_Γ and *Q*_↑_*K* momentum-dark states is removed by the observation of a simultaneous shift of all three peaks upon negative doping at gate voltages below −20 V in Fig. 4b. At this voltage, finite electron population of the Q pockets favors the formation of BL trions with electrons forced to reside in different Q valleys by the Pauli exclusion principle. Thus, all sideband replica associated with *Q*_↑_*K* and *Q*_↑_*Γ* sates are expected to shift simultaneously. This is exactly what we observe upon negative doping with a redshift by the trion binding energy of ~22 meV for all three peaks. The shift of the blue-most peak implies that it originates from the *Q*_↑_*K* rather than the *K*_↓_*Γ* state, as the latter is insensitive to the increasing doping level at Q. Data at higher doping levels and with better signal-to-noise ratio have not been recorded prior to a fatal breakdown of the device and thus a more detailed analysis of charge control of BL WSe_2_ PL must be postponed to future work.

### Quantum dots in bilayer WSe_2_

**Fig. 5:**
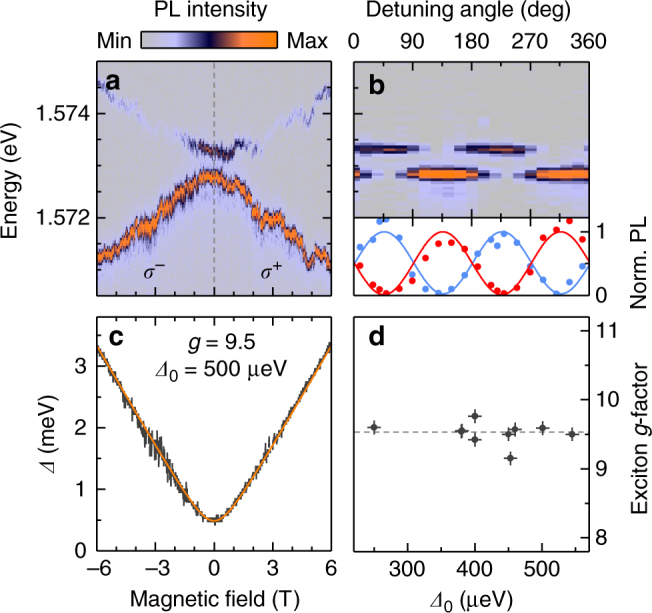
Quantum dots in bilayer WSe_2_. **a** False-color plot of quantum dot magneto-luminescence under *σ*^+^ (*σ*^−^) polarized excitation for positive (negative) magnetic fields in Faraday geometry. **b** The quantum dot emission doublet (upper panel) is characterized by linearly polarized peaks with orthogonal polarization axes (lower panel; note the anti-correlation in the intensities of the higher- and lower-energy peaks shown in red and blue together with squared sine and cosine fits). **c** Energy dispersion of the doublet splitting *Δ* in magnetic field. Best fit to the data with a hyperbolic function (solid line) was obtained for a zero-field fine-structure splitting *Δ*_0_ of 500 μeV and an exciton *g*-factor of 9.5. **d** Distribution of exciton *g*-factors around the mean value of 9.5 plotted for ten quantum dots with respect to their zero-field splitting

The notion of momentum-dark exciton states provides a new perspective on the origin of QDs in ML^42,53–58^ and BL^42,43^ TMDs. In addition to spectrally narrow and bright PL with antibunched photon emission statistics discussed above, BL QDs share all main signatures of localized excitons with ML QDs. In high-resolution micro-PL spectroscopy, they exhibit a doublet of states with orthogonal linear polarization (Fig. 5a, b), which evolves into a pair of circularly polarized Zeeman-split peaks with increasing magnetic field (Fig. 5a, c). The dispersion of the Zeeman splitting *Δ* between the blue and red QD branches with out-of-plane magnetic field *B* according to the hyperbolic function *Δ* = $\sqrt {\left( {g\mu _{\mathrm{B}}B} \right)^2 + {\mathrm{\varDelta }}_0^2}$ (solid line in Fig. 5c) is a hallmark of QDs with anisotropic fine-structure splitting *Δ*_0_^61^. At large enough fields, the linear asymptote of the Zeeman splitting is determined by the exciton *g*-factor scaled by the Bohr magneton *μ*_B_.

By applying this analysis to ten randomly selected QDs on strained BL positions, we extracted *g* and *Δ*_0_ from the hyperbolic fit to the Zeeman splitting as for the QD of Fig. 5a, c with *g* = 9.5 ± 0.1 and *Δ*_0_ = 500 ± 10 μeV. Remarkably, as evidenced from Fig. 5d, the *g*-factor of all ten QDs shows only minor variations around the average value of 9.5 independent of the QD PL energy and despite the spread in the fine-structure splittings in the range of ~400–900 μeV^42,43,53–56,58^. This observation suggests that QD excitons relate to momentum-dark excitons that inherit their *g*-factor from the delocalized continuum state (i.e., *Q*_↑_*Γ* in the case of BL WSe_2_) and exhibit significant brightening due to their spread in momentum space upon spatial localization. This picture is further supported by the sharp cutoff to the emission energy of BL QDs at the energy of *Q*_↑_*Γ* momentum-dark excitons in Fig. 3b as well as in previous studies^42,43^.

For QDs in ML WSe_2_ with similarly sharp cutoff energies at ~20–25 meV below the bright state *X*^42,43,53,55,56,58^ and surprisingly large *g*-factors in the range of 6–12^42,43,53–56^, this insight suggests the presence of a momentum-dark reservoir with energy in between the bright and dark ML excitons *X* and *D* as discussed in ref. ^28^. In ML MoSe_2_ void of momentum-dark states below the bright exciton, on the other hand, no cutoff energy to the QD emission was observed and similar values for the the *g*-factors of QD excitons and the bright exciton *X* were found^57^. To leverage this speculation, theoretical calculations of exciton *g*-factors are required for all excitons constructed from CB electrons and VB unoccupied states in valleys other than K.

### Data availability

The data that support the findings of this study are available from the corresponding author on reasonable request.

## Electronic supplementary material

Supplementary Information
