# Supplementary material for: The role of momentum-dark excitons in the elementary optical response of bilayer WSe2
Source: Nat Commun. 2018 Jul 3;9:2586. doi: 10.1038/s41467-018-04877-3 (PMC6030057; doi:10.1038/s41467-018-04877-3)
Supplement: Supplementary file 1 — Supplementary Information [file 41467_2018_4877_MOESM1_ESM.pdf]

SUPPLEMENTARY INFORMATION

The role of momentum-dark excitons in the elementary optical response of bilayer WSe_2

Jessica Lindlau[1], Malte Selig[2,3], Andre Neumann[1], Léo Colombier[1], Jonathan Förste[1], Victor Funk[1], Michael Förg[1], Jonghwan Kim[4], Gunnar Berghäuser[2], Takashi Taniguchi[5], Kenji Watanabe[5], Feng Wang[4], Ermin Malic[2], and Alexander Högele[1]

[1]*Fakultät für Physik, Munich Quantum Center, and Center for NanoScience (CeNS), Ludwig-Maximilians-Universität München, Geschwister-Scholl-Platz 1, D-80539 München, Germany*

[2]*Chalmers University of Technology, Department of Physics, SE-412 96 Gothenburg, Sweden*

[3]*Institut für Theoretische Physik, Nichtlineare Optik und Quantenelektronik, Technische Universität Berlin, Hardenbergstr. 36, D-10623 Berlin, Germany*

[4]*Department of Physics, University of California at Berkeley, Berkeley, 94720 California, U.S.A.*

[5]*National Institute for Materials Science, Tsukuba, Ibaraki 305-0044, Japan*

Supplementary Note 1: Sample fabrication

The samples of bilayer (BL) WSe_2 on SiO_2 were fabricated by standard mechanical exfoliation from commercially available bulk crystals using polydimethylsiloxane (PDMS) stamping[1]. For the fabrication of gate-tunable samples high quality hexagonal boron nitride (hBN) crystals[2] were used. A first layer of hBN was mechanically exfoliated and transferred onto a Si/SiO_2 (90 nm) substrate using the hot pick-up technique[3]. The deposition of the subsequent monolayer (ML) and the second hBN capping layer was obtained by repeating this procedure. The stack was then placed in contact to a gold pre-patterned electrode. The doping level was varied by applying a bias between the metal electrode and the *p*-doped Si back gate.

Supplementary Note 2: Experimental setup

Confocal spectroscopy studies were performed in liquid helium or a closed-cycle cryostat (attocube systems, attoDRY1000) with base temperatures of 4.2 K and 3.1 K, respectively. The latter cryostat was equipped with a solenoid providing magnetic fields up to ± 9 T. The sample was positioned with piezo steppers and scanners (attocube systems, ANP101 series and ANSxy100/lr) into the diffraction-limited spot of a low-temperature apochromatic objective with a numerical aperture of 0.82 (attocube systems, LT-APO/VISIR/0.82) and a spot size of 0.6 μm in diameter.

A fiber-based home-built microscope, coupled in back scattering geometry to a standard spectrometer (PI, Acton SP-2558) with a nitrogen-cooled silicon CCD (PI, Spec-10:100BR/LN) and a resolution of 0.26 meV (0.05 meV in Fig. 5 of the main text and in Supplementary Fig. 3, bottom panel), was used for photoluminescence (PL), Raman and differential reflectivity (DR) measurements with a supercontinuum laser (NKT Photonics, SuperK EXW-12). Broadband DR spectra were obtained as $(R_{\text{off}} - R_{\text{on}})/R_{\text{off}}$, where R_{off} and R_{on} are the reflectivities off and on the flake. The PL was excited with a continuous wave (cw) diode laser at 639 nm (New Focus, Velocity TLB-6704), a ps-pulsed diode laser at 630 nm (PicoQuant, LDH-P-C-630), or a Ti:sapphire laser (Coherent, Mira 900) operated either in cw or ps-pulsed mode. Photoluminescence excitation (PLE) was performed with the Ti:sapphire laser in cw mode. Two single-photon counting avalanche photo-diodes (PicoQuant, τ-SPAD) were used in a Hanbury Brown-Twiss configuration for measurements of photon statistics.

Supplementary Note 3: Theoretical calculations of exciton dispersions

To compute excitonic binding energies for direct as well as for indirect excitons, we solve the Wannier equation[4,5]

$$\frac{\hbar^2\mathbf{q}^2}{2m}\varphi^\mu_\mathbf{q} - \sum_\mathbf{k} V^{\text{exc}}_{\mathbf{k}-\mathbf{q}}\varphi^\mu_\mathbf{k} + E_{\text{gap}}\varphi^\mu_\mathbf{q} = E^\mu\varphi^\mu_\mathbf{q}. \tag{1}$$

Here, $m = m_\text{e}m_\text{h}/(m_\text{e} + m_\text{h})$ denotes the reduced mass with the electron (empty state) mass m_e (m_h). The latter were obtained from density functional theory calculations for electrons[6] and angle-resolved photoemission spectroscopy measurements for unoccupied states[7]. The electronic bandgap E_gap including band separation for different valleys and spin bands was obtained from density functional theory calculations (electrons)[8] and angle-resolved photoemission spectroscopy experiments (unoccupied states)[7]. The Coulomb matrix element $V^{\text{exc}}_{\mathbf{k}-\mathbf{q}}$ was treated within the Keldysh formalism[9–12], where we can take explicitly into account the dielectric screening from the environment and the width of the investigated transition metal dichalcogenide (TMD) material. Solving the Wannier equation, we have microscopic access to exciton eigenenergies E^μ and excitonic wavefunctions $\varphi^\mu_\mathbf{q}$. Note that we do not include exchange coupling, which is known to lift the degeneracy between spin-like and spin-unlike exciton states[13].

Supplementary Note 4: Peak decomposition procedure

The optical phenomena in TMD BLs derived from the single-particle band structure are shown schematically in Fig. 2a of the main text. The conduction band (CB) and valence band (VB) feature spin-polarized spin-orbit sub-bands at high symmetry points K and K′ of the first Brillouin zone (only the upper valence sub-band is shown in Fig. 2a of the main text). In addition to the K and K′ valleys, the CB exhibits local minima at six non-equivalent Q-pockets related pairwise by time-reversal symmetry[14,15]. In BL TMDs, the energy of the CB at Q shifts well below K and the VB edge at the spin-degenerate Γ point is 40 ± 30 meV below the VB maximum at K[7].

On the basis of this single-particle band structure we construct excitons from an unoccupied state in the upper valence sub-band at K or Γ and the Coulomb-correlated electron at K′, K, or one of the Q-points, as indicated by the dashed ellipses in Fig. 2a of the main text. Neglecting the upper sub-band at Q due to large spin-orbit splitting of the order of 300 meV (see Fig. 2b of the main text) we obtain the exciton configurations of momentum-bright (X and D) and momentum-dark ($Q_\uparrow\Gamma$, $Q_\uparrow K$, $K_\downarrow\Gamma$, $K'_\uparrow K$, $K_\uparrow\Gamma$, and $K'_\downarrow K$) excitons (indicated by dashed lines in Fig. 1c of the main text). Due to their momentum-indirect nature all these excitons do not decay via direct radiative pathways. However, radiative decay can be assisted by acoustic or optical phonons and higher-order phonon processes. The contribution of such phonon-assisted decay to the PL can be obtained as the superposition

	WSe_2		
Mode	Γ	K	Q
TA	0	15.6	11.6
LA	0	18.0	14.3
TO(E′)	30.5	26.7	27.3
LO(E′)	30.8	31.5	32.5
A_1	30.8	31.0	30.4

Supplementary Table 1: Phonon mode energies at the high-symmetry points of the first Brillouin zone for monolayer WSe_2 used in the model fits for bilayer WSe_2 spectra. Phonon modes listed in the table in gray were discarded from the analysis of spectra from samples of bilayer WSe_2 on SiO_2 but included for the spectra of bilayer WSe_2 encapsulated in hexagonal boron nitride and exhibiting spectrally narrow peaks. All energies are given in meV and reproduced from Ref. 16.

of phonon sidebands separated from the bare energy of momentum-dark excitons by the energies of acoustic, optical, or combinations of multiple phonons. In samples with inhomogeneous broadening, the phonon sidebands can be modeled by Gaussians.

The energies of acoustic and optical phonons in ML WSe_2 used in our spectral decomposition procedure are reproduced from Ref. 16 in Supplementary Table 1 for reference. Given the full-width at half-maximum (FWHM) linewidth $\gamma \sim 20$ meV of Gaussians used in our decomposition of spectra from BL WSe_2 on SiO_2, the differences in the energies of acoustic and optical phonons are negligible and thus only one acoustic (the longitudinal acoustic, LA) and one optical (A_1) phonon mode were used. All phonon modes were included in the refined modeling of BL WSe_2 encapsulated in hBN. Note that the phonon energies of Ref. 16 are for ML WSe_2 and we expect deviations for BL WSe_2 of less than ± 2 meV, a value small as compared with the inhomogeneous broadening in both types of samples.

Second- and higher-order phonon-assisted processes were restricted to combinations of multiple phonons with total phonon momentum of Q or K depending on the respective initial valley of the electron. For example, the scattering of an electron from the Q-valley into the K-valley and subsequent emission of an optical phonon would involve an optical or acoustic phonon at the Q-point and a zero-momentum optical phonon at the Γ-point of the first Brilloiun zone. With these considerations, model fits to the lower-energy peak in the experimental BL spectra around 1.55 eV were obtained as contributions from momentum-dark $Q_{\uparrow}\Gamma$, $Q_{\uparrow}K$, and $K_{\downarrow}\Gamma$ excitons which require assistance of acoustic or optical phonons for recombination. The fits were obtained by fixing the

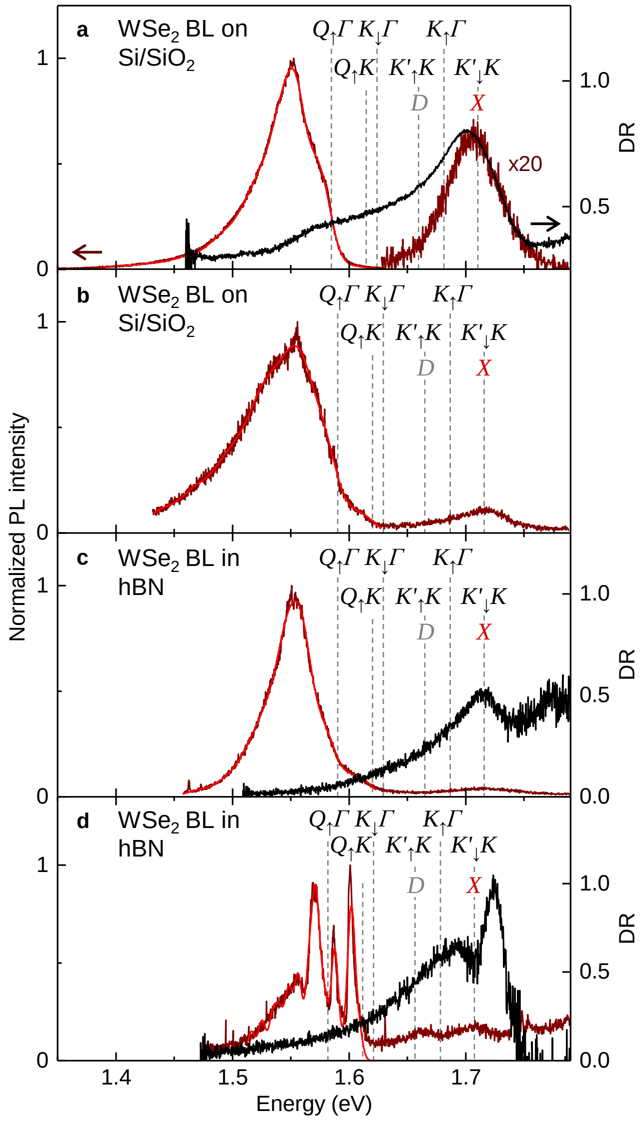

Supplementary Figure 1: Cryogenic photoluminescence and differential reflectivity of WSe_2 bilayers. **a**, Photoluminescence spectrum (brown) with the respective model fit (red) duplicated from Fig. 1c of the main text, and differential reflectivity (black). **b**, Normalized photoluminescence (brown) and model fit (red) for a bilayer WSe_2 on Si/SiO_2 from a different sample. The exciton resonances are blueshifted by 6 meV with respect to **a** as indicated by the dashed lines. **c**, and **d**, Normalized photoluminescence (brown) and differential reflectivity (black) spectra from two different samples of bilayer WSe_2 encapsulated in hexagonal boron nitride; brown and red traces in **d** are reproduced from Fig. 4a of the main text. Best model fits (red) were obtained for inhomogeneous linewidths of 21, 25, 21, and 8 meV in **a**, **b**, **c**, and **d**, respectively.

bare energies of momentum-dark excitons and allowing the fit procedure to determine the amplitudes of Gaussian phonon sidebands with a joint FWHM inhomogeneous linewidth redshifted by the energy of acoustic, optical, or multi-phonon process.

For exciton energies shown in Supplementary Fig. 1a (PL and fit traces same as in Fig. 1c of the main text) and Gaussian phonon sideband amplitudes as free fit parameters, best fit was obtained with a global inhomogeneous linewidth $\gamma = 21$ meV. Same fitting procedure applied to the PL spectrum of another sample of BL WSe_2 on SiO_2 shown in Fig. 1b was obtained with $\gamma = 25$ meV and a global blueshift of momentum-dark exciton energies by 6 meV. The difference in the FWHM linewidths as well as the overall blueshift reflect differences in the effective dielectric environments of BL flakes for the two samples which in turn stem from variations in the exfoliation process. In both cases, second and third order scattering from $K_{\downarrow}\Gamma$ and $Q_{\uparrow}K$, respectively, and up to sixth order scattering from the lowest state $Q_{\uparrow}\Gamma$ were included to reproduce the red-most wing of the PL spectrum. The presence of phonon-scattering processes of such high order out of $Q_{\uparrow}\Gamma$ is consistent with efficient relaxation and trapping of exciton population in this lowest energy momentum-dark state.

We applied the same procedure to model the PL spectra (brown) of two different samples of BL WSe_2 encapsulated in between two layers of hBN (Supplementary Fig. 1c and d; PL traces and fit in d are reproduced from Fig. 4a of the main text). The corresponding DR spectra (black), shown in Supplementary Fig. 1c and d, respectively, highlight interference effects in reflectivity due to multiple reflection planes. To determine the energy position of momentum-indirect excitons referenced to the energy of the bright exciton, we used the respective PL data of each sample and kept all energy spacings constant. Best model fits to the data in Supplementary Fig. 1c and d (red traces) were obtained for inhomogeneous linewidths of 21 meV and 8 meV, respectively. Despite the nominally identical procedures used in the fabrication of the two samples, the difference in the sample quality reflected by the different inhomogeneous broadenings is noticeable but not surprising. These differences in the spectral widths necessitated up to third order phonon scattering in the model fit in Supplementary Fig. 1c and only first (second) order scattering from $K_{\downarrow}\Gamma$ and $Q_{\uparrow}K$ ($Q_{\uparrow}\Gamma$) were required for the model fit in Supplementary Fig. 1d.

Supplementary Note 5: Discussion of PLE spectra and peak ordering

In order to rationalize the energetic ordering of momentum-dark excitons, the data Fig. 3b of the main text is reproduced in both panels of Supplementary Fig. 2. The energies identified from the cut-off to the quantum dot (QD) PL as well as from QD and BL PLE spectra were referenced to the energy of the bright exciton X at 1.710 eV that lies 51 meV above its spin-orbit split momentum-direct spin-dark counterpart D according to theory.

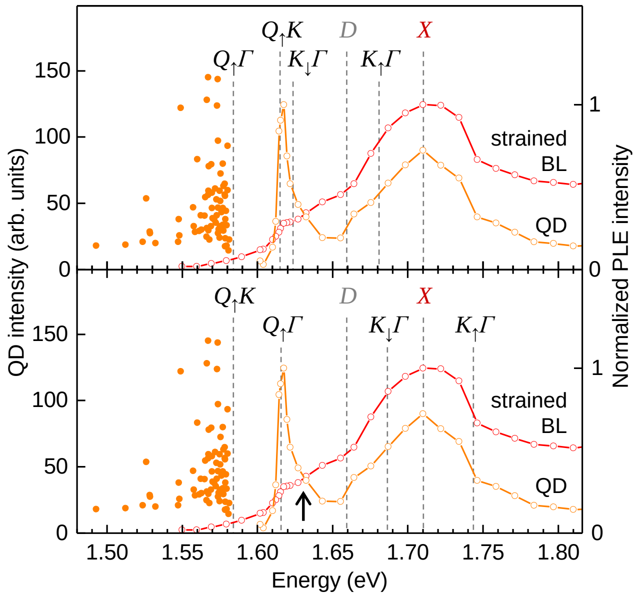

Supplementary Figure 2: Energetic ordering of excitons in bilayer WSe_2. Data duplicated from Fig. 3b of the main text with exciton labeling same as in the main text. Upper panel: energy positions and peak assignments of dark-exciton states according to the theoretically predicted energy scale hierarchy. Lower panel: energy positions of momentum-dark excitons according to the reversed ordering of the two lowest-energy states. Note the missing peak assignment in the lower panel indicated by the arrow.

The energies of the two lowest-energy dark-exciton states were identified as $E_1 = 1.584$ eV and $E_2 = 1.615$ eV, yielding experimental shifts of $\Delta^{\mathrm{exp}}_{1-X} = E_1 - E_X = -126$ meV and $\Delta^{\mathrm{exp}}_{2-X} = E_2 - E_X = -95$ meV with respect to the energy of X. According to theoretical calculations, the two lowest-energy states $Q_{\uparrow}\mathit{\Gamma}$ and $Q_{\uparrow}K$, separated by $\Delta^{\mathrm{th}}_{Q\mathit{\Gamma}-X} = -163$ meV and $\Delta^{\mathrm{th}}_{QK-X} = -161$ meV, respectively, compete for the assignment to the lowest-energy states.

First, we test the scenario of preserved energy scale hierarchy with $Q_{\uparrow}\mathit{\Gamma}$ state being lowest in energy (with energy E_1), followed by the state $Q_{\uparrow}K$ (with energy E_2). We note that the states $Q_{\uparrow}K$ and X share their unoccupied state in the K valley and thus the energy difference can be entirely attributed to the electron in the CB minimum at Q. With $\Delta^{\mathrm{exp}}_{2-X} - \Delta^{\mathrm{th}}_{QK-X} = 66$ meV, we thus directly obtain the upshift of the exciton energy with the unoccupied state in K and the electron in Q. For the state $Q_{\uparrow}\mathit{\Gamma}$ we obtain the difference between the experimental and theoretical values as $\Delta^{\mathrm{exp}}_{1-X} - \Delta^{\mathrm{th}}_{Q\mathit{\Gamma}-X} = 37$ meV, which implies a downshift of the VB maximum at Γ by 29 meV by using the upshift of the CB minimum at Q calculated above. The energy of the state $K_{\uparrow}\mathit{\Gamma}$, which shares with X the electron in the spin-up polarized sub-band at K, computes by including the downshift of Γ to 1.681 eV.

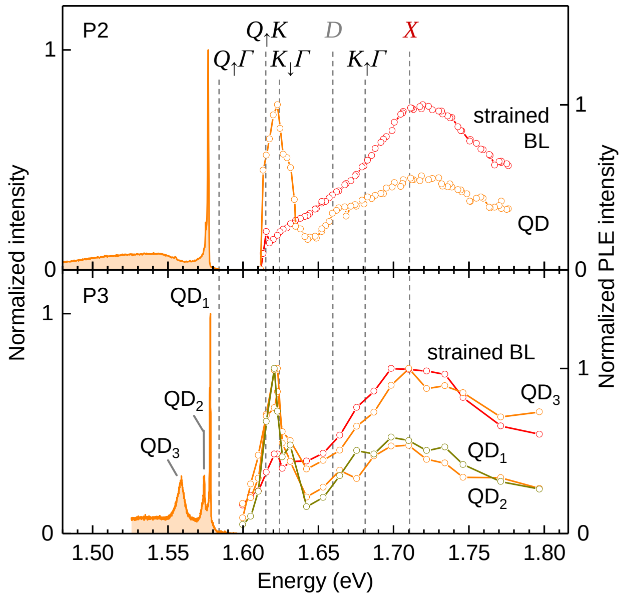

Supplementary Figure 3: Photoluminescence excitation of quantum dots and strained bilayers. Same as in Supplementary Fig. 2 but recorded on different positions (P2 top, P3 bottom) of disorder-strained bilayer WSe_2 with quantum dot emission. The exciton energies marked by the dashed lines are the same as in the upper panel of Supplementary Fig. 2 and in Fig. 3b of the main text.

Finally, the energy of the state $K_{\downarrow}\Gamma$ is obtained as 1.624 eV by taking into account the theoretically calculated spin-orbit splitting of 57 meV between $K_{\uparrow}\Gamma$ and $K_{\downarrow}\Gamma$.

The second scenario probes the reversed ordering where the state $Q_{\uparrow}K$ is lowest (with energy E_1) and $Q_{\uparrow}\Gamma$ is second-lowest state (with energy E_2). Calculations of the respective energies for all relevant momentum-dark states along the lines of arguments given above yields an upshift of the Q valley by 35 meV and an upshift of the Γ valley by 33 meV. Accordingly, the energies of $K_{\uparrow}\Gamma$ and $K_{\downarrow}\Gamma$ states are obtained as 1.743 eV and 1.686 eV, respectively.

The energy positions for all relevant excitons obtained from the two competing assignment scenarios are plotted as dashed lines in Supplementary Fig. 2. The upper panel shows the energetic ordering in accord with preserved hierarchy, while the lower panel shows the results of reversed ordering. The failure of the latter to predict the resonance in PLE at 1.624 eV (indicated by the black arrow in the lower panel) which is consistently ascribed in the framework of the former to the state $K_{\downarrow}\Gamma$, provides strong evidence for $Q_{\uparrow}\Gamma$ being the lowest and $Q_{\uparrow}K$ being the second-lowest state.

Remarkably, all PLE resonances appear at the same energy positions for different BL regions subjected to unintentional strain (red PLE spectra in Supplementary Fig. 3) with confocal PL from QDs emitting at different PL energies (orange and dark green PLE spectra in Supplementary Fig. 3). This observation indicates that the PLE resonances are not QD-specific (e.g. due to excited QD states that would differ from dot to dot because of different confinement potentials) but indeed probe the absorption of BL WSe_2.

Supplementary References

1. Castellanos-Gomez, A. *et al.* Deterministic transfer of two-dimensional materials by all-dry viscoelastic stamping. *2D Mater.* **1**, 011002 (2014).

2. Taniguchi, T. & Watanabe, K. Synthesis of high-purity boron nitride single crystals under high pressure by using Ba-BN solvent. *J. Cryst. Growth* **303**, 525–529 (2007).

3. Pizzocchero, F. *et al.* The hot pick-up technique for batch assembly of van der Waals heterostructures. *Nat. Commun.* **7**, 11894 (2016).

4. Kira, M. & Koch, S. Many-body correlations and excitonic effects in semiconductor spectroscopy. *Prog. Quant. Electron.* **30**, 155–296 (2006).

5. Haug, H. & Koch, S. W. *Quantum Theory of the Optical and Electronic Properties of Semiconductors* 5*th edn.* (World Scientific Publishing, 2009).

6. Wickramaratne, D., Zahid, F. & Lake, R. K. Electronic and thermoelectric properties of few-layer transition metal dichalcogenides. *J. Chem. Phys.* **140**, 124710 (2014).

7. Wilson, N. R. *et al.* Determination of band offsets, hybridization, and exciton binding in 2D semiconductor heterostructures. *Sci. Adv.* **3**, 1601832 (2017).

8. Terrones, H. & Terrones, M. Bilayers of transition metal dichalcogenides: Different stackings and heterostructures. *J. Mater. Res.* **29**, 373–382 (2014).

9. Keldysh, L. V. Coulomb interaction in thin semiconductor and semimetal films. *JETP Lett.* **29**, 658 (1978).

10. Cudazzo, P., Tokatly, I. V. & Rubio, A. Dielectric screening in two-dimensional insulators: Implications for excitonic and impurity states in graphane. *Phys. Rev. B* **84**, 085406 (2011).

11. Berghäuser, G. & Malic, E. Analytical approach to excitonic properties of MoS_2. *Phys. Rev. B* **89**, 125309 (2014).

12. Schmidt, R. *et al.* Ultrafast Coulomb-induced intervalley coupling in atomically thin WS_2. *Nano Lett.* **16**, 2945–2950 (2016).

13. Qiu, D. Y., Cao, T. & Louie, S. G. Nonanalyticity, valley quantum phases, and lightlike exciton dispersion in monolayer transition metal dichalcogenides: Theory and first-principles calculations. *Phys. Rev. Lett.* **115**, 176801 (2015).

14. Zhao, W. *et al.* Evolution of electronic structure in atomically thin sheets of WS_2 and WSe_2. *ACS Nano* **7**, 791–797 (2013).

15. Liu, H. *et al.* Observation of intervalley quantum interference in epitaxial monolayer tungsten diselenide. *Nat. Commun.* **6**, 8180 (2015).

16. Jin, Z., Li, X., Mullen, J. T. & Kim, K. W. Intrinsic transport properties of electrons and holes in monolayer transition-metal dichalcogenides. *Phys. Rev. B* **90**, 045422 (2014).
